# Supplementary material for: Are improper kinetic models hampering drug development?
Source: PeerJ. 2014 Oct 28;2:e649. doi: 10.7717/peerj.649 (PMC4217195; doi:10.7717/peerj.649)
Supplement: Supplemental Information 2 [file peerj-02-649-s002.docx]

$$v=V_{1}\frac{[S]}{[S]+K_{1}}-V_{1}\frac{[S]^{H1}}{[S]^{H1}+K_{2}^{H1}}+V_{2}\frac{[S]^{H1}}{[S]^{H1}+K_{2}^{H1}}-V_{2}\frac{[S]^{H2}}{[S]^{H2}+K_{3}^{H2}}$$

Terms used to define the production of β-amyloid by γ-secretase (equation 5):

*v = reaction rate*

*S = substrate*

*V_1_ = maximum reaction rate at low substrate concentrations*

*V_2_ = maximum reaction rate at high substrate concentrations*

*K_1_ = substrate affinity term at low substrate concentrations*

*K_2_ = substrate affinity term at high substrate concentrations*

*K_3_ = substrate affinity term at high substrate concentrations which produce inhibition*

*H1 = Hill coefficient used to define the substrate activation slope*

*H2 = Hill coefficient used to define the substrate inhibition slope*

$$V_{1}=V_{1}-\left(V_{1}-V_{1is}\right)\frac{[I]^{Hx1}}{[I]^{Hx1}+K_{x1}^{Hx1}}+\left(V_{1}-V_{1is}\right)\frac{[I]^{Hx2}}{[I]^{Hx2}+K_{x2}^{Hx2}}-\left(V_{1}-V_{1ii}\right)\frac{[I]^{Hx2}}{[I]^{Hx2}+K_{x2}^{Hx2}}$$

Terms used to define DAPT modification of the V_1_ term:

*V_1is_ =increase in reaction rate produced by low DAPT concentrations*

*V_1ii_ =decrease in reaction rate produced by high DAPT concentrations*

*K_x1_ = DAPT affinity binding term at low DAPT concentrations*

*K_x2_ = DAPT affinity binding term at high DAPT concentrations*

*Hx1 = Hill coefficient used to define the DAPT activation slope*

*Hx2 = Hill coefficient used to define the DAPT inhibition slope*

$$K_{1}=K_{1}-\left(K_{1}-K_{1is}\right)\frac{[I]^{Hx1}}{[I]^{Hx1}+K_{x1}^{Hx1}}+\left(K_{1}-K_{1is}\right)\frac{[I]^{Hx2}}{[I]^{Hx2}+K_{x2}^{Hx2}}-\left(K_{1}-K_{1ii}\right)\frac{[I]^{Hx2}}{[I]^{Hx2}+K_{x2}^{Hx2}}$$

Terms used to define DAPT modification of the K_1_ term:

*K_1is_ =change in substrate affinity term produced by low DAPT concentrations*

*K_1ii_ = change in substrate affinity term produced by high DAPT concentrations*

*K_x1_ , K_x2_, Hx1 and Hx2 are the same as above.*

$$V_{2}=V_{2}-\left(V_{2}-V_{2i}\right)\frac{[I]^{Hxx}}{[I]^{Hxx}+K_{xx1}^{Hxx}}$$

Terms used to define DAPT modification of the V_2_ term:

*V_2i_ = decrease in reaction rate produced by DAPT*

*K_xx1_ = DAPT affinity binding term*

*Hxx = Hill coefficient used to define the DAPT inhibition slope*

$$K_{2}=K_{2}-\left(K_{2}-K_{2i}\right)\frac{[I]^{Hxx}}{[I]^{Hxx}+K_{xx1}^{Hxx}}$$

Terms used to define DAPT modification of the K_2_ term:

*K_2i_ = change in substrate affinity term produced by DAPT concentrations*

*K_xx1_ and Hxx are the same as above.*
