# Supplementary figures and images for: Are improper kinetic models hampering drug development?
Source: PeerJ. 2014 Oct 28;2:e649. doi: 10.7717/peerj.649 (PMC4217195; doi:10.7717/peerj.649)

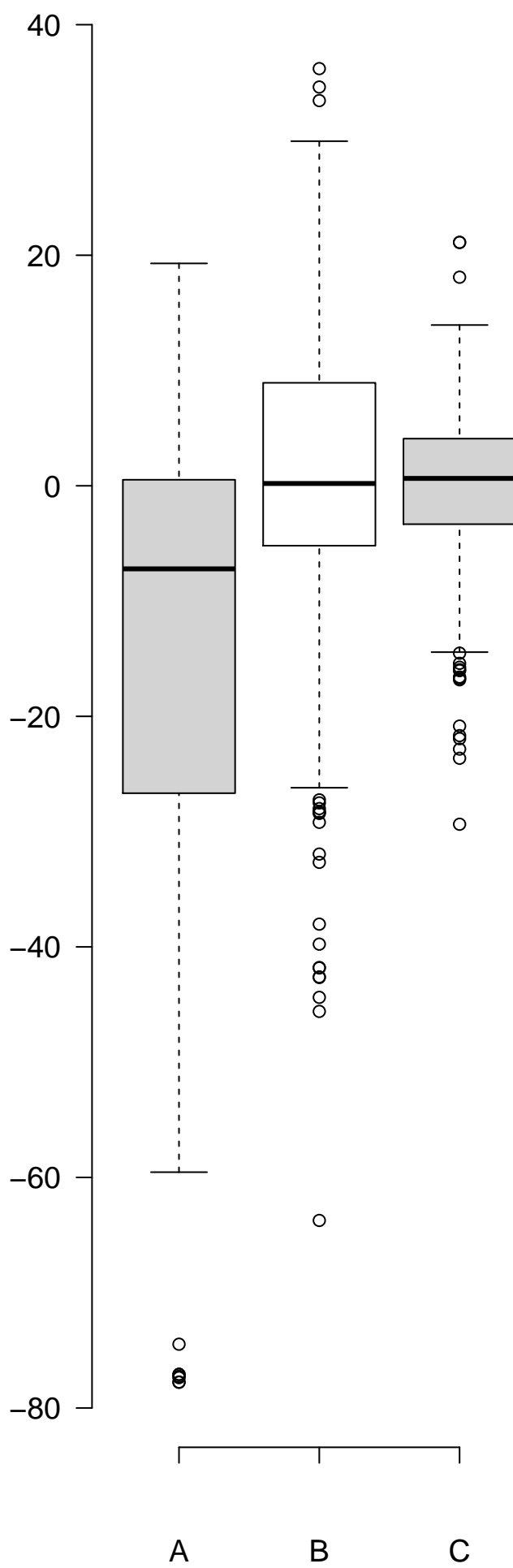

Supplement: Figure S1 — (A) Residuals produced by Eq. (10) with the published values, (B) residuals associated with the Eq. (10) after it was refit to the data and (C) residuals associated with Eq. (5). Center lines show the medians; box limits indicate the 25th and 75th percentiles as determined by R software; whiskers extend 1.5 times the interquartile range from the 25th and 75th percentiles; outliers are represented by dots. Since Eq. (10) was not fit to background substrate concentrations, for A and B, n = 208, while for C (Eq. (5)) n = 230 sample points. This plot was generated using the web-tool BoxplotR (Spitzer et al., 2014). [file peerj-02-649-s003.pdf]
